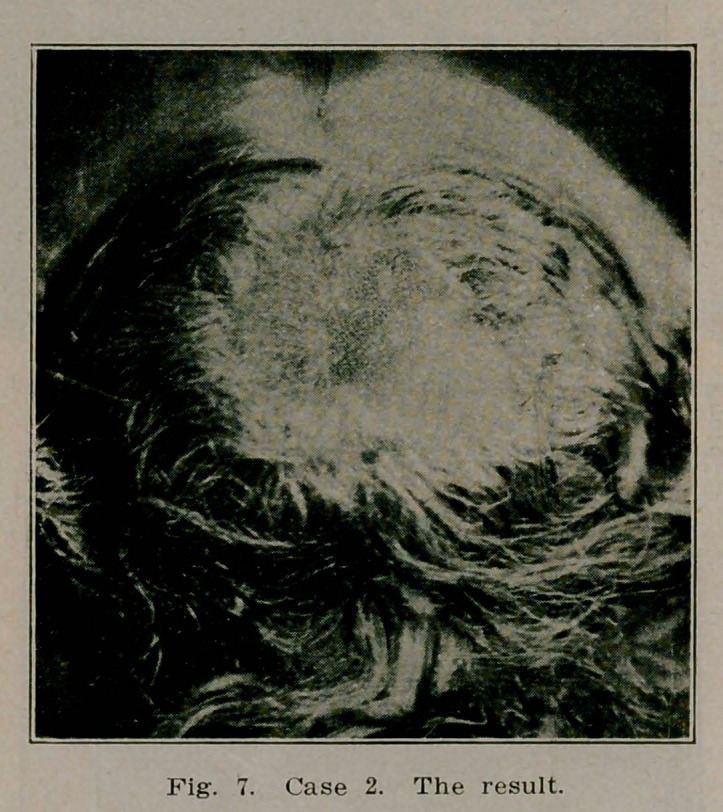# Nonsurgical Treatment of Cancer vs. Surgical

**Published:** 1917-02

**Authors:** 


**Nonsurgical Treatment of Cancer vs. Surgical.** G. N. Murphy, Paducah, Ky., Med. Summary, Oct., quotes McFarland as having operated on 118 cases with 2 cures, Samuel Gross as having operated on 408 cases of cancer of the breast with 1 cure, all the rest dying of recurrence within 3 years, D. Hayes Agnew as having removed enough cancerous breasts to fill a cart without saving a single life. Murphy claims between 300 and 400 cases treated without the knife and the saving of 80-85%. Cuts by courtesy of author and editor.

## Figures and Tables

**Fig. 1. f1:**
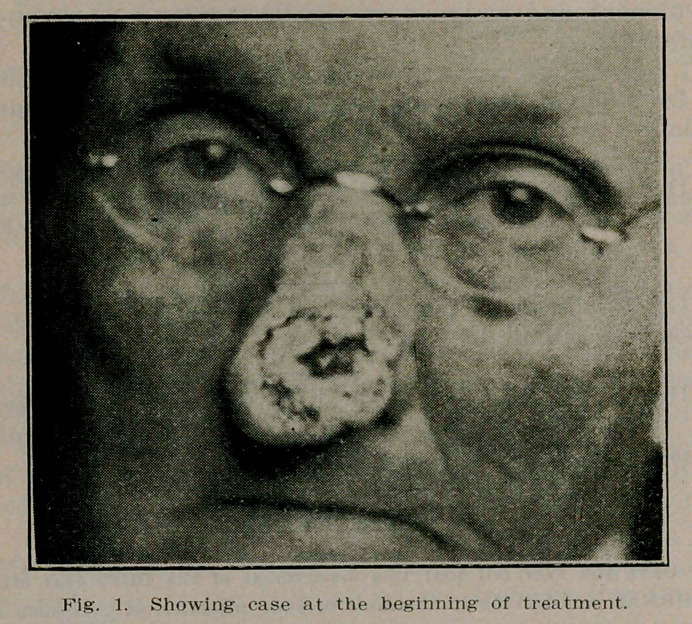


**Fig. 2. f2:**
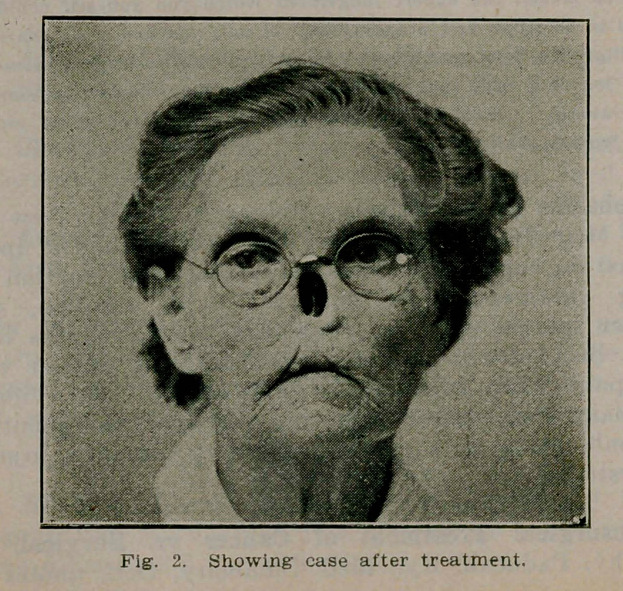


**Fig. 3. f3:**
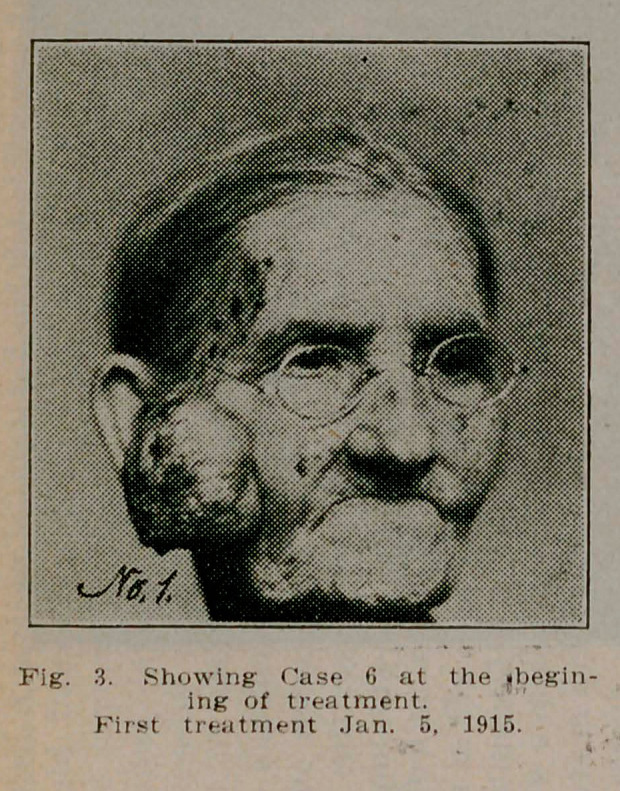


**Fig. 4. f4:**
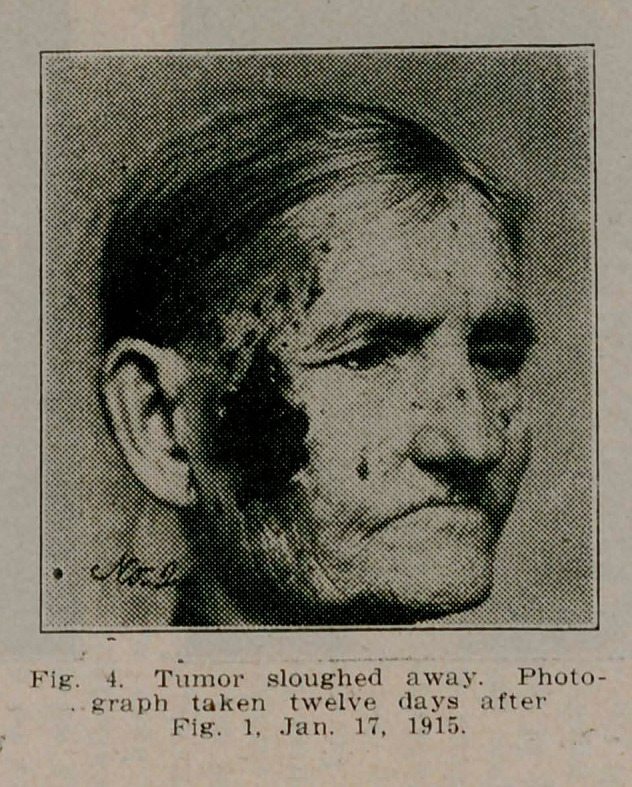


**Fig. 5. f5:**
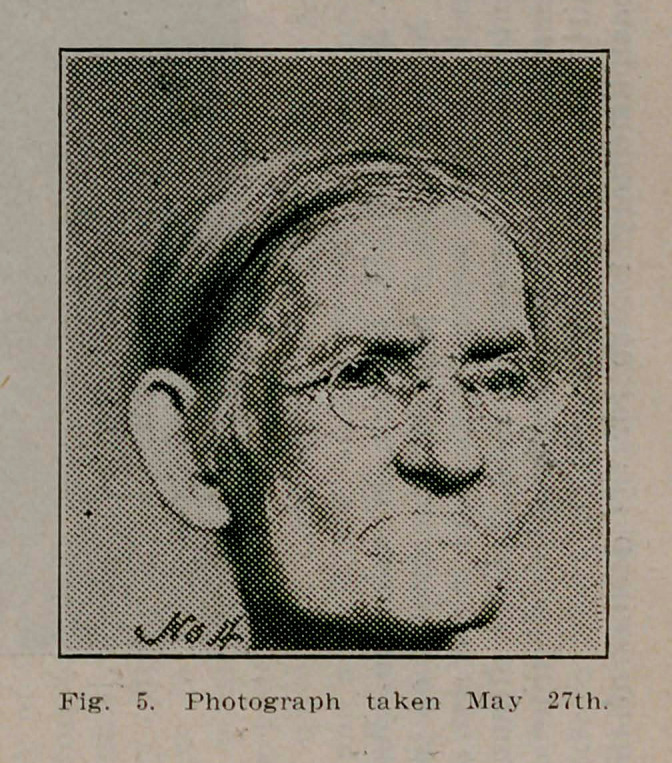


**Fig. 6. f6:**
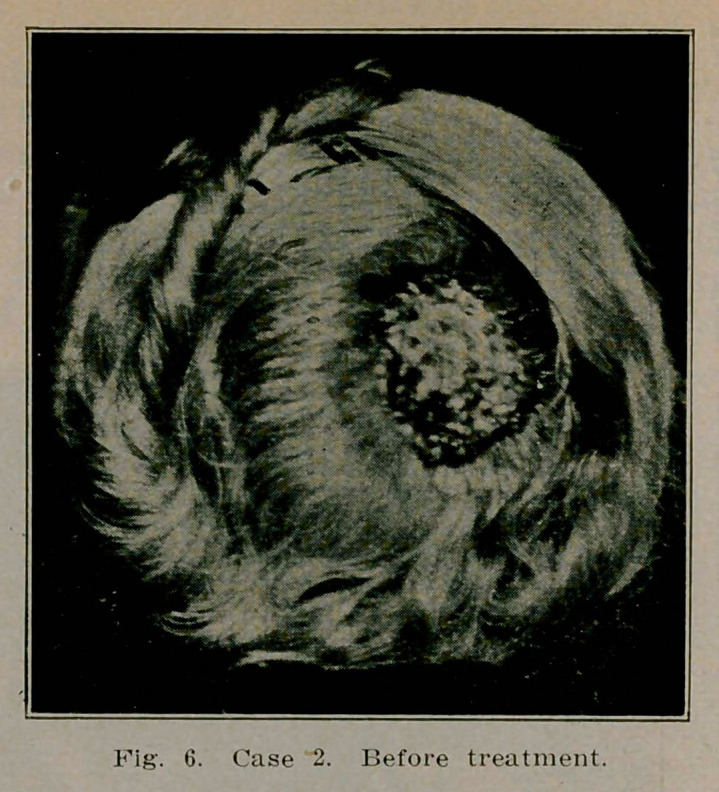


**Fig. 7. f7:**